# Fabry Disease Screening in Patients with Idiopathic HCM or LVH: Data from the Multicentric Nationwide F-CHECK Study

**DOI:** 10.3390/biomedicines13102530

**Published:** 2025-10-16

**Authors:** Raquel Machado, Inês Fortuna, Sílvia Sousa, Catarina Costa, João Calvão, Ana Filipa Amador, Patrícia Rodrigues, Dulce Brito, Marta Vilela, Natália António, Vanessa Lopes, Cristina Gavina, Ana Sofia Correia, Conceição Queirós, Alexandra Toste, Alexandra Sousa, Ricardo Fontes-Carvalho, André Lobo, Inês Silveira, Janete Quelhas-Santos, Elisabete Martins

**Affiliations:** 1 Faculty of Medicine, University of Porto, 4200-319 Porto, Portugal; romachado@med.up.pt (R.M.); ifasilva@med.up.pt (I.F.); silviasousa@med.up.pt (S.S.); cgavina@med.up.pt (C.G.); fontes.carvalho@gmail.com (R.F.-C.); sjanete@med.up.pt (J.Q.-S.); 2 Department of Cardiology, Centro Hospitalar Universitário de São João, European Reference Network for Rare, Low-Prevalence, or Complex Diseases of the Heart (ERN GUARD-Heart), 4200-219 Porto, Portugal; catarinamarcosta@gmail.com (C.C.); joaocalvao1@gmail.com (J.C.); a.filipa.amador@gmail.com (A.F.A.); 3 Department of Cardiology, Centro Hospitalar Universitário do Porto, 4050-342 Porto, Portugal; pfdrodrigues@gmail.com; 4 Unit for Multidisciplinary Research in Biomedicine, Institute for the Biomedical Sciences Abel Salazar, University of Porto, 4050-313 Porto, Portugal; 5 Department of Cardiology, Hospital Universitário de Santa Maria, 1649-028 Lisboa, Portugal; dulcebrito59@gmail.com (D.B.); martamiguezvilela@gmail.com (M.V.); 6 CCUL@RISE, Faculty of Medicine, University of Lisbon, 1649-028 Lisboa, Portugal; 7 Department of Cardiology, Centro Hospitalar e Universitárrio de Coimbra, 3004-561 Coimbra, Portugal; natalia.antonio@gmail.com (N.A.); vlopes.pt@gmail.com (V.L.); 8 Faculty of Medicine, University of Coimbra, 3000-548 Coimbra, Portugal; 9 Department of Cardiology, ULS Matosinhos, 4464-513 Matosinhos, Portugal; sofiakorreia@gmail.com; 10 Department of Cardiology, ULS Tâmega e Sousa, 4564-007 Penafiel, Portugal; superpluto@sapo.pt; 11 Hospital da Luz, 1500-650 Lisboa, Portugal; alexandra.toste6@gmail.com; 12 Department of Cardiology, ULS Entre Douro e Vouga, 4520-211 Santa Maria da Feira, Portugal; xanasousa81@gmail.com; 13 Department of Cardiology, ULS Gaia e Espinho, 4434-502 Vila Nova de Gaia, Portugal; andresmplobo@gmail.com; 14 Department of Cardiology, ULS Trás-os-Montes e Alto Douro, 5000-508 Vila Real, Portugal; ines.c.silveira@gmail.com

**Keywords:** Fabry disease, screening, cardiomyopathies, left ventricular hypertrophy

## Abstract

**Background/Objectives**: Fabry disease (FD) is a rare X-linked disease caused by the deficient activity of the enzyme α-galactosidase A. Cardiac involvement is particularly critical, often determining the disease prognosis. Epidemiological data on FD in Portugal are limited and inconsistent, highlighting the need for targeted screening. The F-CHECK study aimed to determine the prevalence of FD through the systematic screening of a Portuguese cohort of patients with unexplained cardiomyopathies. **Methods**: This multicenter observational study (NCT05409846) assessed the prevalence and clinical characteristics of FD in a Portuguese cohort (*n* = 409) of patients from 10 central hospitals who presented with unexplained cardiomyopathies, including idiopathic hypertrophic cardiomyopathy (HCM), left ventricular hypertrophy, dilated-phase HCM, and dilated cardiomyopathy with late gadolinium enhancement in the inferolateral segment. Screening was performed using dried blood spot assays to measure α-galactosidase A activity and/or by GLA gene sequencing in whole-blood samples. **Results**: FD was diagnosed in 14 patients, corresponding to a prevalence of 3.4%. FD diagnosis was significantly associated with systemic manifestations such as acroparesthesias (*p* = 0.027) and angiokeratomas (*p* = 0.003), as well as an increased risk of prior arrhythmic events (*p* = 0.021) and cerebrovascular disease (*p* = 0.016). Most FD patients (57%) presented a non-founder mutation in the GLA gene; however, they were pathogenically relevant. **Conclusions**: The observed 3.4% prevalence highlights the importance of systematic FD screening among Portuguese patients with unexplained cardiomyopathy, extending beyond classic hypertrophic presentations to dilated forms. Specific clinical signs, electrocardiogram findings, and cardiac imaging features can serve as valuable indicators to guide targeted genetic testing for FD.

## 1. Introduction

Fabry disease (FD) is a rare X-linked lysosomal storage disease (LSD) caused by mutations in the *GLA* gene, which encodes the enzyme alpha-galactosidase A (α-Gal A). The deficiency or absence of α-Gal A leads to the intracellular accumulation of globotriaosylceramide and related glycosphingolipids within lysosomes across various cell types and organ systems, including the kidneys, heart, and nervous systems [1]. The accumulation triggers a complex cascade of pathophysiological processes, such as cellular hypertrophy, fibrosis, and inflammation, that ultimately cause progressive organ damage, life-threatening complications, and increased risk of premature death [2,3].

Clinically, FD is categorised into two major phenotypes: classic and later-onset. The classic phenotype is characterised by severely reduced (<3% of normal values) or absent α-Gal A activity and typically presents in early childhood with signs and symptoms such as cornea *verticillata*, acroparesthesias, and angiokeratomas [1,2]. Over time, patients often develop progressive multi-organ involvement, including chronic kidney disease with proteinuria, leading to end-stage renal failure, hypertrophic cardiomyopathy (HCM), sensorineural hearing loss, and cerebrovascular events. In contrast, the later-onset phenotype occurs in individuals with residual enzyme activity and usually lacks early symptoms. Clinical manifestations are often milder, delayed, or confined to a single organ, most commonly the heart, where significant pathology such as left ventricular hypertrophy (LVH) may emerge later in life [1].

Cardiac involvement is the primary determinant of morbidity and mortality in FD [3,4]. Multiple cardiac cell types may be affected, resulting in various cardiac phenotypes and clinical presentations. While concentric HCM is the most frequent cardiac manifestation, other variants such as asymmetric LVH, apical HCM, or even systolic left ventricular (LV) dysfunction may occur. Clinical consequences include heart failure (HF), arrhythmias, and ischemic events [1,5]. Given the progressive nature of FD and the availability of disease-specific therapies, early and accurate clinical diagnosis is critical for optimising treatment outcomes. Notably, studies have shown that enzyme replacement therapy (ERT) offers limited benefit when initiated after age 40 or in patients with advanced cardiac involvement, including significant LVH or established myocardial fibrosis [1].

Genetic testing is essential for diagnosing FD in males and females [1]. In males with the classic phenotype, a diagnosis can typically be confirmed by demonstrating severely reduced or absent α-Gal A activity [1,5]. DBS shows high sensitivity but lower specificity for FD in males, easily identifying true positives but also increasing the likelihood of false positives [6]. As such, is necessary to confirm positive cases with genetic analysis of the *GLA* gene. In females, diagnosis is more complex due to X-chromosome inactivation and variable expression. Enzyme activity may fall within the normal range, making genetic analysis of the *GLA* gene necessary for confirmation [5]. FD has been identified in approximately 0.5% to 1% of patients diagnosed with HCM, although distinguishing it from more common sarcomeric HCM forms remains challenging [3]. Due to FD’s heterogeneous and often nonspecific presentation, the systematic screening of patients with compatible phenotypes is considered the most effective approach to improve diagnostic accuracy.

In Portugal, data on FD prevalence remain limited but indicate a potentially significant disease burden, particularly in certain regions. A 2004 study by Pinto et al. estimated the national birth prevalence of LSDs in the country, identifying four FD cases diagnosed between 1982 and 2001, corresponding to a prevalence of 0.5% in the northern region of Portugal and 2.0% in other areas [7]. More recently, a Portuguese multicentre screening study conducted between 2008 and 2018, involving 780 patients with HCM, reported an FD prevalence of 4.7%, primarily attributed to the p.F113L founder variant [8]. In contrast, the Portuguese Registry of HCM (PRo-HCM), which included 1042 patients from 29 centre between 2013 and 2015, found no GLA pathogenic variants among the 528 patients who underwent genetic testing [9]. These conflicting findings underscore the importance of systematic and targeted screening to clarify FD epidemiology in Portugal and improve diagnostic precision. Table 1 summarises several FD screening studies in patients with cardiomyopathies.

This study aims to screen for FD in patients presenting with a range of cardiac phenotypes, specifically those with cardiomyopathy of unknown or uncertain aetiology. The objectives are to facilitate the timely diagnosis of FD, enhance understanding of the disease’s national epidemiology, and raise awareness among clinicians managing patients whose clinical presentations may be attributable to FD.

## 2. Materials and Methods

The F-CHECK study was a multicentre, observational epidemiological study, conducted between January 2021 and January 2025, enrolling 409 patients referred from cardiomyopathy consultation across 10 Portuguese hospitals (NCT05409846). Patient recruitment commenced in April 2022, after obtaining ethical approval. Patients were not involved in the design, or conduct, or reporting, or dissemination plans of our research.

Inclusion criteria encompassed patients diagnosed with: idiopathic HCM, defined by LV wall thickness ≥ 15 mm (Group A); idiopathic LVH with wall thickness ≥ 13 mm (Group B); the dilated phase of HCM (Group C); and dilated cardiomyopathy of unknown aetiology, with late gadolinium enhancement (LGE) on cardiac magnetic resonance (CMR) affecting the inferolateral basal segment (Group D). Patients were diagnosed according to the 2023 ESC guidelines for the management of cardiomyopathies [19].

**Figure 1:**
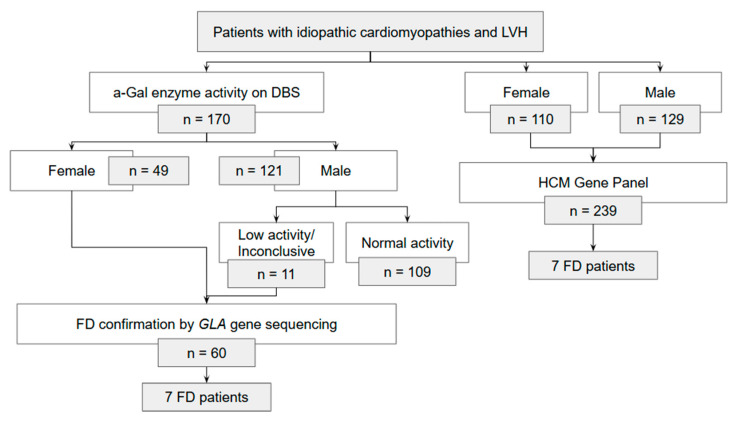
Flowchart scheme of Fabry disease (FD) diagnosis in patients with idiopathic cardiomyopathies and left ventricular hypertrophy (LVH).

Initially, DBS samples were collected for enzymatic analysis of α-Gal A activity, regardless of sex. Genetic testing of the *GLA* gene was performed in all female participants, in males with reduced α-Gal A activity, and in individuals with inconclusive enzymatic results or without prior DBS testing. The study protocol was originally designed to align with contemporary clinical practice in Portugal, in which DBS testing was routinely ordered prior to genetic analysis. Over the course of the study period, however, this practice evolved, and DBS testing was progressively discontinued in favour of direct genetic testing with HCM panels. To ensure adequate sample size, some participants who had undergone only genetic testing were therefore included. The flowchart in Figure 1 provides a detailed overview of the number of patients included through each pathway.

Genetic testing was performed by accredited diagnostic laboratories (ISO 15189) [20] using validated next-generation sequencing (NGS) cardiomyopathy panels, which included complete coverage of the *GLA* gene. Copy-number variation (CNV) analysis of *GLA* was performed. All variants classified as pathogenic or likely pathogenic were confirmed by Sanger sequencing before clinical reporting. Variant classification and description were performed by the certified laboratories, following international recommendations (PMID: 25741868, 23887774, 21681106), reducing subjective classification bias. Population frequencies were verified using gnomAD and DGV, and variant classification was conducted according to ACMG guidelines.

Sociodemographic and clinical data were collected from electronic clinical records, including cardiovascular history, current and past symptoms and signs, cardiovascular risk factors, and medication use. Additionally, the most recent findings from electrocardiogram (ECG), Holter monitoring, echocardiography, and CMR were analysed.

This project was approved by the Ethics Committee for Health of CHUSJ (CE/409/21) and other centres, according to the principles of the Helsinki Declaration, the Convention on Human Rights and Biomedicine, and the guidelines of the Council for International Organisations of Medical Sciences, and written informed consent was obtained from each patient.

No formal sample size calculation or power analysis was performed a priori. Statistical analyses were performed using R version 4.4.2 (R Foundation for Statistical Computing). Variables are presented as median and interquartile range (IQR). Due to the small number of FD cases, only non-parametric tests were used for group comparisons. The Mann–Whitney U test was applied to continuous variables, while categorical variables were analysed using the appropriate chi-square test or Fisher’s exact test (expected frequency < 5 in any cell). Statistical significance was defined as *p* < 0.05. Missing values were reported explicitly in tables using “-“ where data were not available. No imputation was performed. Analyses were conducted using available-case data for each variable.

Associations between FD and clinical or imaging features were assessed using logistic regression. For the overall cohort, unadjusted and multivariable-adjusted odds ratios (ORs) with 95% confidence intervals (CIs) were calculated. Adjusted models included age, sex, relevant comorbidities and medication use to account for potential confounding. Subgroup analyses were performed by LV phenotype (hypertrophic and dilated), using unadjusted logistic regression due to the limited number of FD patients in each subgroup.

The study included all consecutive patients meeting inclusion criteria during the recruitment period across participating centres to minimise sources of bias. Consequently, the sample size was determined by patient availability rather than statistical considerations. The relatively small number of FD cases, particularly in subgroup analyses, is acknowledged as a limitation and discussed in terms of precision and interpretability of results.

This study is reported in accordance with the STROBE guidelines for observational studies, and a completed STROBE checklist is provided in the Supplementary Materials (Supplemental Table S1).

## 3. Results

A total of 409 patients were enrolled in the study, including 250 males (61%), with a median age of 64 (range: 18–93) years. Group A comprised 72% of patients (*n* = 293), group B included 10% of patients (*n* = 78), group C accounted for 7.3% (*n* = 30), and group D for 2.0% (*n* = 8). FD was diagnosed in 14 patients, corresponding to an observed prevalence of genetically confirmed FD of 3.4% (95% CI [1.9, 5.7]) Among the 170 patients who underwent DBS testing, 71% were male (Figure 1). Of these, reduced α-Gal A activity was detected in 9% (n = 11). All female patients who underwent DBS and male patients with reduced enzyme activity were evaluated with *GLA* gene sequencing (n = 60). Seven *GLA* variants were identified in these patients (12%). Additionally, 239 patients underwent genetic testing alone, with pathogenic *GLA* variants identified in seven cases (2.9%). The full variant metadata is available in Appendix A Table A1.

### 3.1. Demographics and Clinical Characteristics of Patients

The demographic and clinical characteristics of FD and non-FD patients are summarised in Table 2. No significant differences were observed between FD and non-FD patients regarding age, sex, and body mass index (BMI). However, certain clinical features were significantly more common among FD patients, such as acroparesthesias (14 vs. 1.5%, *p* = 0.027) and angiokeratomas (14 vs. 0.3%, *p* = 0.003). FD patients had a significantly higher prevalence of prior arrhythmic events compared to non-FD patients (36 vs. 12%, *p* = 0.021). Cerebrovascular disease was more frequently observed in FD patients (29 vs. 6.9%, *p* = 0.016). Additionally, FD patients were less often prescribed β-blockers than non-FD patients (43% vs. 71%, *p* = 0.033), while anticoagulant use was significantly higher in the FD group (50% vs. 23%, *p* = 0.049).

### 3.2. Characteristics of Patients with FABRY DISEASE

FD patients had a median age of 63 (IQR 59, 65) years, and 57% were male (*n* = 8). The median BMI was 27 (25, 29) kg/m^2^. Cardiovascular symptoms/signs were present in 86% of patients (*n* = 12), with fatigue being the most frequently reported symptom, affecting 57% (*n* = 8). At least one comorbidity or cardiovascular risk factor was present in 86% (*n* = 12), with dyslipidaemia being the most common, observed in 57% of patients (*n* = 8), followed by hypertension, present in 50% (*n* = 7). Previous cardiovascular events were documented in 50% of FD patients (*n* = 7), with arrhythmias (primarily atrial fibrillation), being the most common, occurring in 36% (*n* = 4).

Among the 14 patients with genetically confirmed FD, seven distinct pathogenic mutations in the *GLA* gene were identified. The most frequent was the p.F113L variant, found in 43% (*n* = 6), followed by p.M290I in 21% (*n* = 3) and p.N215S in 14% (*n* = 2). The remaining four patients carried other distinct *GLA* gene variants. The characteristics of these 14 patients are summarised in Table A2. Some demographic and known FD red flags in patients with classical phenotype variants and patients with late-onset variants are summarised in Table A3.

### 3.3. Comparing FD Patients with Non-FD in Different Cardiac Phenotypic Spectra

When patients were stratified by LV phenotype, hypertrophic (groups A and B) versus dilated (groups C and D), FD was more frequent in patients with a dilated phenotype than those with a hypertrophic phenotype.

A comparison of ECG and Holter characteristics between FD and non-FD participants is presented in Table 3, dividing the patients according to LV phenotype. When comparing FD patients with non-FD patients, FD patients showed a significantly longer QRS duration in both the hypertrophic group (130 vs. 103 ms, *p* = 0.029) and the dilated group (169 vs. 112 ms, *p* = 0.013). However, the proportion of patients with a prolonged QRS duration (>110 ms) did not reach statistical significance between groups. FD patients in the hypertrophic group also had a higher prevalence of right bundle branch block (RBBB) compared to non-FD patients (56 vs. 9.1%, *p* < 0.001) and fascicular block (33% vs. 7.8%, *p* = 0.033). These ECG differences were also present in the dilated group but did not reach statistical significance.

In the 24 h Holter monitoring, FD patients in the hypertrophic group had significantly higher mean heart rates (HRs) than non-FD (89 vs. 66 bpm, *p* = 0.018). Maximum and minimum HR values were also higher in the hypertrophic FD patients; however, in the dilated group, FD patients had lower HRs than non-FD patients. These differences may reflect variations in medication use.

Table 4 compares imaging characteristics between FD and non-FD participants according to LV phenotype. Echocardiographic assessment revealed that FD patients had a smaller left atrial (LA) diameter than non-FD patients, with a more pronounced and statistically significant difference in the dilated group (39 vs. 48 mm, *p* = 0.013). Regarding LV function, FD patients in the hypertrophic group demonstrated a statistically significant trend toward lower LV ejection fraction (LVEF) than non-FD patients (57 vs. 62%, *p* = 0.042). In contrast, LVEF was higher in FD patients in the dilated group, although this difference was not statistically significant. While interventricular septum (IVS) thickness did not significantly differ between groups, the prevalence of IVS hypertrophy (IVS > 12 mm) was lower in FD patients in the hypertrophic group compared to non-FD patients (60 vs. 88%, *p* = 0.027).

In the CMR assessment, the only statistically significant difference was the presence of LGE in the inferolateral basal segment, which was more frequently observed in FD patients than in non-FD patients in the hypertrophic group (78 vs. 19%, *p* < 0.001).

### 3.4. Clinical and Cardiac Outcomes According to Diagnosis and Ventricular Phenotype

Logistic regression analyses were performed to evaluate associations between FD and clinical as well as imaging features (Table 5). For the overall cohort, both unadjusted and multivariable-adjusted ORs with 95% CIs were calculated, adjusting for age, sex, comorbidities, and medication use. Subgroup analyses stratified by LV phenotype (hypertrophic vs. dilated) were performed using unadjusted ORs only, due to the limited number of FD patients in each subgroup, focusing on exam-derived variables (ECG, echocardiography, and CMR parameters).

In the overall cohort, FD was associated with higher odds of arrhythmia (adjusted OR 4.6 [1.2, 17.6]), acroparesthesias (adjusted OR 12.6 [1.3, 97.9]), angiokeratomas (OR 65.5 [5.9, 1469]), and cerebrovascular disease (adjusted OR 5.8 [1.3, 21.6]). Other clinical outcomes, including heart failure, cardiac device use, and common symptoms showed non-significant trends.

Among the hypertrophic subgroup, FD patients demonstrated higher unadjusted odds of RBBB (OR 13.5 [3.1, 53.1]), fascicular block (OR 5.9 [1.2, 24.0]), and LGE in the inferolateral segment (OR 14.5 [3.4, 100]), while IVS hypertrophy was less frequent (OR 0.2 [0.1, 0.8]). In the dilated subgroup, OR estimates were generally less precise due to very small numbers, with wide confidence intervals.

These findings highlight that FD is associated with specific clinical and imaging features overall, with certain ECG and CMR abnormalities particularly pronounced in the hypertrophic phenotype. The subgroup analyses should be interpreted cautiously given the limited sample sizes.

## 4. Discussion

The importance of screening for FD in patients with cardiac involvement is well established, and several studies have examined its prevalence in different cohorts over the past years. Reported prevalence among patients with unexplained cardiomyopathy or LVH varies across international studies, reflecting differences in genetic backgrounds, inclusion criteria, and screening methodologies. A prospective study conducted in Edmonton and Hong Kong identified FD in 2.0% of patients (5/266) with undiagnosed LVH [21]. Similarly, two large Chinese cohorts reported prevalence rates of 0.9% (8/906) [22] and 1.8% (11/602) [23] among patients with LVH and HCM, respectively. In Europe, a multicentre study applying strict inclusion criteria (men aged ≥35 years and women aged ≥40 years with unexplained LVH) found a lower prevalence of 0.5% (7/1386) [12]. Of these, two patients had the p.N215S mutation, two had the p.A143T variant, and the remaining three had distinct mutations (p.R118C, p.D244N, and p.T410A). Additional studies in Spain [10] and the Czech Republic [17] reported prevalence rates of 1.0% (5/508 and 6/589, respectively) among HCM patients, with recurrent identification of later-onset variants such as p.N215S and p.A143T. In Portugal, the burden of FD appears to be higher, attributed mainly to the p.F113L founder mutation [8]. However, data from the PRo-HCM showed no *GLA* mutations among 528 genotyped patients [9]. These contrasting findings highlight the need for more targeted and systematic screening strategies to avoid underdiagnosis, particularly in regions with known founder effects. In our cohort, we identified a prevalence of 3.4% for FD, reinforcing the relevance of such strategies and contributing valuable epidemiological data to the national context. Notably, most studies to date have focused exclusively on patients with unexplained hypertrophic phenotypes, often neglecting those with a dilated LV. In our cohort, 29% of patients diagnosed with FD presented with a dilated cardiac phenotype, although the limited sample, it emphasises the need to broaden screening criteria beyond classical HCM presentations to capture the full clinical spectrum of FD-related cardiac disease.

The different methodologies used for FD screening should also be considered. DBS testing, though useful, has limitations due to its low specificity, particularly in females and in patients with late-onset variants, and often requires genetic confirmation. Nonetheless, it remains a valuable tool in settings with limited access to genetic testing. The growing availability of cardiomyopathy gene panels that include *GLA* as a standard gene has improved diagnostic efficiency and facilitated earlier identification of FD. However, identifying a *GLA* variant necessitates careful classification of pathogenicity, a process that is not always straightforward and may evolve as evidence accumulates. Monda et al. conducted a systematic review and meta-analysis to assess how the classification of *GLA* gene variants affects the estimated prevalence of FD in cardiac screening studies [24]. The results highlighted inconsistencies in variant interpretation across studies and the risk of overestimating FD prevalence if non-pathogenic variants are misclassified, as reclassification of variants, especially p.A143T, p.D313Y, and p.E66Q, significantly influenced prevalence estimates. Our cohort identified seven distinct mutations, each requiring consideration within a clinical and population-specific context. The p.F113L variant remains the most well-characterised in the Portuguese population, a known founder mutation. Additionally, the p.M290I variant has been described in the Madeira population [25], and the p.R118C mutation has been linked to increased stroke risk in Portuguese patients [26]. The identified mutations represent a broad spectrum of clinical phenotypes, ranging from classic and severe forms of FD to late-onset presentations, and even variants whose pathogenicity remains controversial or uncertain. This distribution highlights the considerable phenotypic heterogeneity inherent in cardiac FD.

Early recognition of FD is paramount for preventing irreversible organ damage and facilitating the timely initiation of specific therapies, such as ERT, which can significantly slow disease progression. The profound implications for patient management underscore the critical need for accurate *GLA* variant pathogenicity assignment. The identification of seven distinct *GLA* gene mutations in a cohort of 409 cardiomyopathy patients, yielding an approximate prevalence of 3.4%, suggests that a substantial number of FD cases within cardiac populations may remain undiagnosed through conventional clinical pathways or broader screening efforts. This finding reveals a notable genetic contribution of FD to this specific cardiac patient population, underscoring the diagnostic yield of focused genetic investigations in high-risk clinical settings.

Consequently, cardiologists must adopt a highly nuanced diagnostic approach, recognising that FD is a spectrum disorder. Relying solely on the classic presentation will inevitably lead to significant underdiagnosis, necessitating a broader suspicion for various cardiac and non-cardiac symptoms. Furthermore, for several specific mutations there is a geographical dimension to variant prevalence (e.g., p.F113L in Portugal/Southern Italy). Therefore, cardiologists and genetic counsellors in specific geographic regions should be particularly cognizant of the prevalence of specific *GLA* variants within their local or ancestral populations. This regional awareness can refine diagnostic suspicion and guide more targeted screening strategies, potentially improving diagnostic efficiency and patient identification in specific ethnic or geographical groups.

Well-established clinical red flags of FD were also observed in our cohort. Features such as acroparesthesias and angiokeratomas were significantly more prevalent among FD patients than non-FD individuals (*p* = 0.027 and *p* = 0.003, respectively). The clinical suspicion of FD frequently arises from the coexistence of these extracardiac manifestations along with certain features of cardiac disease. For example, disproportionate conduction abnormalities, early arrhythmic events, or stroke in a patient with LVH should prompt consideration of FD. Furthermore, FD patients exhibited increased prior arrhythmic events cerebrovascular disease (*p* = 0.016), highlighting the systemic nature of the disease and the importance of multidisciplinary recognition of these warning signs.

Although our study was limited by the relatively small number of patients with genetically confirmed FD, we conducted a subgroup exploratory analysis to compare FD and non-FD participants according to LV phenotype. FD patients demonstrated significantly longer QRS durations than non-FD patients in both the hypertrophic (*p* = 0.029) and dilated (*p* = 0.013) subgroups. In the hypertrophic group, FD patients presented more frequently with RBBB (*p* < 0.001) and fascicular block (*p* = 0.033). These findings are consistent with the literature, highlighting conduction system involvement as a hallmark of cardiac FD [2]. Interestingly, these differences persisted in the dilated phenotype, although they did not achieve statistical significance, likely due to the small number of patients in this subgroup. Echocardiographic analysis further revealed that FD patients had smaller LA diameters than non-FD patients, with the difference reaching statistical significance in the dilated group (*p* = 0.013). This aligns with prior studies comparing FD and HCM patients, in which FD was associated with smaller LA volumes [27]. However, existing literature focuses predominantly on the hypertrophic phenotype, and our findings offer new insight into LA remodelling in the context of the dilated presentation of FD, an area previously uncharacterised. Regarding LV systolic function, FD patients in the hypertrophic group exhibited a significantly lower LVEF than their non-FD counterparts (*p* = 0.042), suggesting early contractile impairment or less frequent supranormal ejection compared to other HCM aetiologies [28]. Conversely, in the dilated group, FD patients tended to have relatively higher values of LVEF. IVS thickness did not differ significantly between groups; however, FD patients in the hypertrophic subgroup were less likely to present with septal hypertrophy (*p* = 0.027). This finding may support the hypothesis that LVH in FD follows a different remodeling pattern, often with less prominent septal involvement than in sarcomeric HCM. On cardiac MRI, LGE in the inferolateral basal segment was more frequent in FD patients within the hypertrophic group than non-FD patients (*p* < 0.001). This specific LGE distribution is well recognized in FD and supports its diagnostic value. However, the role of this feature in the diagnosis of FD in patients with dilated phenotype is still unclear.

In addition to the comparative analyses, logistic regression was performed to further explore the association between FD and specific clinical, ECG, and imaging features. In the overall cohort, both unadjusted and adjusted models were applied, with adjustment for age, sex, hypertension, dyslipidemia, B-blocker, and anticoagulant use. In the subgroup analyses, unadjusted models were employed due to the limited number of FD cases within each LV phenotype. The regression results demonstrated that several features, such as arrhythmia, cerebrovascular disease, acroparesthesias, and angiokeratomas, were significantly associated with FD in the overall analysis. Within the hypertrophic and dilated subgroups, conduction abnormalities, particularly right bundle branch block and fascicular block, were strongly associated with FD, corroborating the descriptive findings. Conversely, LV hypertrophy and LGE inferolateral distribution exhibited distinct trends depending on the LV phenotype, suggesting differing structural remodeling mechanisms across disease stages. Nevertheless, the wide CIs observed across several ORs reflect the small number of FD patients, introducing statistical uncertainty despite consistent effect directions. In some models, ORs could not be reliably estimated due to zero events or sparse data, underscoring the challenges of regression analysis in rare diseases. These findings highlight the need for larger, multicentric studies to validate the identified associations and refine the phenotypic predictors of FD across the hypertrophic and dilated spectrum.

## 5. Conclusions

In conclusion, our findings underscore the clinical relevance of systematic screening for FD in patients with unexplained cardiac phenotypes, including both hypertrophic and dilated presentations. The observed prevalence of 3.4% in our cohort, along with distinct ECG and imaging features (by echocardiography and CMR), reinforces the need to integrate FD into the differential diagnosis of cardiomyopathies. The inclusion of the *GLA* gene in cardiomyopathy genetic panels, combined with heightened clinical suspicion based on cardiac and extracardiac red flags, may enhance early diagnosis and appropriate management. However, the interpretation of genetic variants remains complex and context-dependent, requiring continued efforts in variant classification and population-specific characterisation. Our results contribute novel insights into the phenotypic variability of cardiac FD, particularly in the underexplored dilated phenotype, and highlight the importance of comprehensive, multidisciplinary approaches to improve detection and care for affected individuals.

## Figures and Tables

**Table 1:** Fabry disease cardiomyopathy: global screening summary.

Reference/Country	Study Design/Method	Participant/FD Prevalence	Mutations
Monserrat, 2007 [10]/Spain	Plasma α-Gal A activity + *GLA* gene testing in 508 unrelated patients with HCM	508/5 (1.0%)	p.E358del, p.S238N, p.A143T and others
Havndrup, 2010 [11]/Denmark	*GLA* gene testing in probands without sarcomere-gene mutations	59/3 (5.0%)	p.A156T, p.G271S and p.N139S
Elliot, 2011 [12]/Europe	HPLC α-Gal A mutation screening + *GLA* gene testing in LVH patients	1386/7 (0.5%)	p.N215S, p.R118C, p.A143T and others
Hagège, 2011 [13]/France	DBS α-Gal A activity + genotyping in HCM patients	392/4 (1.5%)	p.W162C, p.F113L, and p.N215S
Terryn, 2013 [14]/Belgium	DBS α-Gal A activity + *GLA* gene testing in LVH patients	540/6 (0.9%)	p.A143T
Mawatari, 2013 [15]/Japan	Serum and leukocyte α-Gal A activity + *GLA* gene testing in males with LVH	738/3 (0.4%)	p.E66Q
Cardim, 2018 [9]/Portugal	National HCM registry	528/-	-
Azevedo, 2020 [8]/Portugal	DBS α-Gal A activity + *GLA* gene testing in HCM patients	780/37 (4.7%)	p.F113L, p.C94S, p.M290I and others
Citro, 2022 [16]/Italy	DBS α-Gal A activity + *GLA* gene testing in LVH patients with FD red flags	30/3 (10%)	p.R301G and p.F113L
Zemánek, 2022 [17]/Czech Republic	DBS α-Gal A activity and lyso-Gb3 + *GLA* gene testing in HCM patients	589/6 (1.0%)	p.N215S and p.L294*
Leung, 2024 [18]/China	DBS α-Gal A activity + *GLA* gene testing in LVH patients	426/3 (0.7%)	p.M290T and IVS4+919G>A

DBS, dried blood spot; FD, Fabry disease; HCM, hypertrophic cardiomyopathy; HPLC, high-performance liquid chromatography; LVH, left ventricular hypertrophy.

**Table 2:** Demographic and clinical characteristics of Fabry disease (FD) and non-FD patients.

	Non-FD (*n* = 395)	FD (*n* = 14)	*p* Value
Age, median (IQR)	64 (55, 71)	63 (59, 65)	0.356
18–40 years	35 (8.9%)	0 (0%)	0.067
41–65 years	177 (45%)	11 (79%)	
>65 years	180 (46%)	3 (21%)	
Male sex	242 (61%)	8 (57%)	0.785
BMI (kg/m^2^), median (IQR)	28 (25, 31)	27 (25, 29)	0.607
Study Group			0.032
Dilated	34 (8.6%)	4 (29%)	
Hypertrophic	361 (91%)	10 (71%)	
Symptoms	275 (70%)	11 (79%)	0.568
Fatigue	193 (49%)	8 (57%)	0.596
Dyspnea	76 (19%)	3 (21%)	0.740
Palpitations	59 (15%)	2 (14%)	-
Syncope	31 (7.9%)	0 (0%)	-
Chest Pain	71 (18%)	2 (14%)	-
Murmur	69 (18%)	3 (21%)	0.721
Edema	23 (5.8%)	2 (14%)	0.209
Acroparesthesias	6 (1.5%)	2 (14%)	0.027
Angiokeratomas	1 (0.3%)	2 (14%)	0.003
Abdominal pain	2 (0.5%)	1 (7.1%)	0.100
Family History	126 (32%)	7 (50%)	0.244
Risk Factors	314 (80%)	12 (86%)	0.745
Diabetes	85 (22%)	2 (14%)	0.743
Hypertension	250 (63%)	7 (50%)	0.399
Dyslipidaemia	199 (51%)	8 (57%)	0.787
Smoking	110 (28%)	6 (43%)	0.235
Carpal Tunnel Syndrome	14 (3.6%)	0 (0%)	-
*Cornea verticillate*	0 (0%)	1 (7.1%)	-
Cerebrovascular Disease	27 (6.9%)	4 (29%)	0.016
Kidney Disease	38 (9.6%)	0 (0%)	-
Medication	347 (89%)	10 (71%)	0.075
ACE/ARA	129 (33%)	3 (21%)	0.563
B-blockers	280 (71%)	6 (43%)	0.033
Spironolactone	34 (8.7%)	0 (0%)	-
Diuretic	96 (24%)	2 (14%)	0.533
Aspirin	47 (12%)	1 (7.1%)	-
Anticoagulants	91 (23%)	7 (50%)	0.049
Amiodarone	14 (3.6%)	0 (0%)	-
ARNI	76 (19%)	3 (21%)	0.741
iSGLT2	50 (13%)	1 (7.1%)	-
Previous CV events	134 (34%)	7 (50%)	0.256
Heart Failure	42 (11%)	2 (14%)	0.655
Arrhythmia	46 (12%)	5 (36%)	0.021
Cardiac Devices	42 (11%)	4 (29%)	0.061
Heart Surgery	15 (3.8%)	0 (0%)	-
Cardioembolic	33 (8.4%)	0 (0%)	-

ACE/ARA, angiotensin-converting enzyme inhibitors and angiotensin-receptor antagonists; ARNI, angiotensin receptor-neprilysin inhibitor; BMI, body mass index; CV, cardiovascular; FD, Fabry disease; iSGLT2, sodium-glucose transport protein inhibitors; SD, standard deviation. For categorical variables, Fisher’s exact test was used when expected cell counts were <5.

**Table 3:** Electrocardiogram (ECG) and Holter monitoring characteristics in Fabry disease (FD) and non-FD patients according to left ventricular phenotype.

	Hypertrophic Group			Dilated Group		
	Non-FD (*n* = 361)	FD (*n* = 10)	*p* Value	Non-FD (*n* = 34)	FD (*n* = 4)	*p* Value
ECG						
Heart Rate (bpm) median (IQR)	64 (58, 73)	74 (66, 77)	0.235	72 (55, 78)	59 (57, 62)	0.199
Left Atrial Anomaly	31 (10%)	1 (11%)	-	5 (20%)	0 (0%)	-
LVH–Voltage	98 (32%)	4 (44%)	0.476	5 (20%)	0 (0%)	-
LVH–Overload	87 (28%)	2 (22%)	-	7 (28%)	1 (33%)	-
NVRA	133 (43%)	4 (44%)	-	8 (32%)	1 (33%)	-
Atrioventricular Block	35 (11%)	0 (0%)	-	1 (4.0%)	1 (25%)	0.261
QRS Duration (ms) median (IQR)	103 (94, 114)	130 (110, 148)	0.029	112 (102, 136)	169 (167, 189)	0.013
QRS > 110 ms	90 (31%)	3 (60%)	0.332	13 (50%)	3 (100%)	0.232
LBBB	21 (6.8%)	0 (0%)	-	4 (16%)	0 (0%)	-
RBBB	28 (9.1%)	5 (56%)	<0.001	3 (12%)	2 (67%)	0.073
Pathological Q Waves	28 (9.1%)	0 (0%)	-	6 (24%)	0 (0%)	-
Fascicular block	24 (7.8%)	3 (33%)	0.033	2 (8.0%)	1 (33%)	0.298
Holter Monitoring						
HR mean (bpm) median (IQR)	66 (61, 73)	89 (83, 95)	0.017	72 (61, 81)	62 (61, 63)	0.443
HR maximum (bpm) median (IQR)	106 (96, 121)	130 (124, 130)	0.124	108 (98, 120)	86 (85, 87)	0.056
HR minimum (bpm) median (IQR)	47 (42, 52)	55 (43, 64)	0.217	46 (41, 55)	43 (40, 45)	0.395

bpm, beats per minute; ECG, electrocardiogram; FD, Fabry disease; HR, heart rate; LBBB, left bundle branch block; LVH, left ventricular hypertrophy; NVRA, nonspecific ventricular repolarization abnormalities; RBBB, right bundle branch block. For categorical variables, Fisher’s exact test was used when expected cell counts were <5.

**Table 4:** Echocardiogram and cardiac magnetic resonance (CMR) characteristics in Fabry disease (FD) and non-FD patients according to left ventricular phenotype.

	Hypertrophic Group			Dilated Group		
	Non-FD (*n* = 361)	FD (*n* = 10)	*p* Value	Non-FD (*n* = 34)	FD (*n* = 4)	*p* Value
Echocardiogram						
LA diameter (mm) median (IQR)	43 (39, 48)	40 (38, 41)	0.149	48 (44, 53)	39 (37, 40)	0.013
LA volume (mL/m^2^) median (IQR)	42 (33, 49)	36 (30, 37)	0.106	46 (41, 60)	40 (31, 48)	0.565
LVS diameter (mm) median (IQR)	30 (26, 37)	31 (28, 45)	0.723	45 (38, 53)	43 (NA)	-
LVD diameter (mm) median (IQR)	45 (39, 49)	45 (43, 51)	0.484	54 (51, 61)	55 (NA)	-
LVEF (%) median (IQR)	62 (58, 66)	57 (56, 60)	0.042	42 (34, 49)	52 (49, 55)	0.139
IVS thickness (mm) median (IQR)	16 (14, 19)	16 (12, 18)	0.357	16 (13, 18)	15 (12, 18)	0.761
IVS hypertrophy (>12 mm)	302 (88%)	6 (60%)	0.027	23 (77%)	3 (75%)	-
PW thickness (mm) median (IQR)	11 (10, 13)	13 (11, 15)	0.075	12 (10, 14)	10 (10, 13)	0.780
LVOTO	116 (35%)	4 (40%)	0.745	9 (30%)	1 (25%)	-
CMR						
LA area (cm^2^) median (IQR)	28 (24, 33)	28 (25, 30)	0.751	31 (26, 40)	29 (24, 34)	0.601
LVESV (mL/m^2^) median (IQR)	27 (19, 36)	28 (25, 41)	0.375	50 (41, 57)	44 (43, 44)	0.548
LVEDV (mL/m^2^) median (IQR)	75 (65, 88)	77 (69, 85)	0.945	100 (92, 113)	103 (89, 104)	0.834
LVEF (%) median (IQR)	65 (59, 72)	65 (56, 69)	0.469	49 (36, 56)	55 (48, 58)	0.518
RVESV (mL/m^2^) median (IQR)	22 (17, 29)	23 (22, 31)	0.326	25 (20, 44)	15 (13, 18)	0.115
RVEDV (mL/m^2^), median (IQR)	65 (56, 77)	73 (61, 81)	0.224	71 (61, 81)	54 (52, 56)	0.147
RVEF (%) median (IQR)	67 (61, 73)	64 (61, 70)	0.518	54 (48, 66)	71 (66, 77)	0.210
LV mass (g/m^2^) median (IQR)	84 (65, 101)	83 (73, 103)	0.753	95 (78, 109)	119 (111, 127)	0.210
Papillary muscle hypertrophy	49 (20%)	1 (11%)	-	1 (3.8%)	0 (0%)	-
LV hypertrabeculation	8 (3.3%)	0 (0%)	-	1 (3.8%)	0 (0%)	-
LGE	208 (78%)	9 (90%)	0.696	26 (84%)	3 (100%)	-
LGE inferolateral	40 (19%)	7 (78%)	<0.001	13 (50%)	1 (33%)	-

FD, Fabry disease; IVS, interventricular septum; LA, left atrium; LGE, late gadolinium enhancement; LV, left ventricle; LVD, left ventricle diastolic; LVEDV, left ventricle end-diastolic volume; LVEF, left ventricular ejection fraction; LVESV, left ventricle end-systolic volume; LVOTO, left ventricular outflow tract obstruction; LVS, left ventricle systolic; PW, posterior wall; RVEDV, right ventricle end-diastolic volume; RVEF, right ventricular ejection fraction; RVESV, right ventricle end-systolic volume. For categorical variables, Fisher’s exact test was used when expected cell counts were <5. NA (not applicable): not enough observations to calculate IQR.

**Table 5:** Odds ratios (ORs) for clinical and cardiac features associated with Fabry disease.

Outcome	Overall Unadjusted OR (95% CI)	Overall Adjusted * OR (95% CI)	Hypertrophic OR (95% CI)	Dilated OR (95% CI)
Heart Failure	1.4 [0.2, 5.4]	2.1 [0.3, 9.1]		
Arrhythmia	4.2 [1.3, 12.7]	4.6 [1.2, 17.6]		
Cardiac Devices	3.4 [0.9, 10.5]	3.5 [0.8, 12.5]		
Fatigue	1.4 [0.5, 4.3]	1.5 [0.5, 4.9]		
Dyspnea	1.1 [0.3, 3.8]	1.5 [0.3, 5.3]		
Palpitations	0.9 [0.1, 3.6]	1.1 [0.2, 4.5]		
Chest Pain	0.8 [0.1, 2.9]	0.8 [0.1, 3.1]		
Murmur	1.3 [0.3, 4.2]	1.3 [0.3, 4.7]		
Acroparesthesias	10.8 [1.5, 52.8]	12.6 [1.3, 97.9]		
Angiokeratomas	65.5 [5.9, 1469]	-		
Cerebrovascular Disease	5.4 [1.4, 17.5]	5.8 [1.3, 21.6]		
LVH voltage			1.7 [0.4, 6.6]	-
QRS > 110 ms			3.2 [0.5, 25.1]	-
RBBB			13.5 [3.1, 53.1]	14.6 [1.1, 382]
Fascicular block			5.9 [1.2, 24.0]	5.8 [0.2, 99]
IVS hypertrophy			0.2 [0.1, 0.8]	0.9 [0.1, 20.1]
LGE inferolateral			14.5 [3.4, 100]	0.5 [0.0, 5.9]

* adjusted for age, sex, comorbidities (hypertension and dyslipidaemia) and medication (B-blockers and anticoagulants). Cells marked with “-“ indicate that ORs could not be reliably calculated, typically due to zero events in one group or insufficient sample size, resulting in unstable or undefined estimates.

## Data Availability

The data presented in this article are not readily available due to privacy and ethical reason. Requests for access to these data should be made to the corresponding author, Elisabete Martins, who can be reached at ebernardes@med.up.pt.

## Supplementary Materials

The following supporting information can be downloaded at: https://www.mdpi.com/article/10.3390/biomedicines13102530/s1. Table S1. STROBE Statement—checklist of items that should be included in reports of observational studies.

## Abbreviations

The following abbreviations are used in this manuscript:

BMI	Body mass index
CMR	Cardiac magnetic resonance
DBS	Dried blood spot
ECG	Electrocardiogram
ERT	Enzyme replacement therapy
FD	Fabry disease
HCM	Hypertrophic cardiomyopathy
HF	Heart failure
HR	Heart rates
IQR	Interquartile range
IVS	Interventricular septum
LA	Left atrium
LGE	Late gadolinium enhancement
LSD	Lysosomal storage disease
LV	Left ventricle
LVD	Left ventricle diastolic
LVEDV	Left ventricle end-diastolic volume
LVEF	Left ventricular ejection fraction
LVESV	Left ventricle end-systolic volume
LVH	Left ventricular hypertrophy
LVOTO	Left ventricular outflow tract obstruction
LVS	Left ventricle systolic
OR	Odds Ratio
PW	Posterior wall
RBBB	Right bundle branch block
RVEDV	Right ventricle end-diastolic volume
RVEF	Right ventricular ejection fraction
RVESV	Right ventricle end-systolic volume
SD	Standard deviation
α-GAL A	α-galactosidase A

## Appendix A

**Table A1:** Summary of GLA gene variants detected in patients with Fabry disease, including HGVS nomenclature, ClinVar accession numbers, and pathogenicity classification according to ACMG/ClinVar criteria.

Variant (HGVS)	ClinVar Accession (VCV)	Population Frequency (gnomAD)	ACMG Classification
NM_000169.3(GLA):c.337T>C (p.Phe113Leu)	VCV000222218.26	Not available	Pathogenic
NM_000169.3(GLA):c.916C>T (p.Gln306Ter)	VCV000198052.6	Not available	Pathogenic
NM_000169.3(GLA):c.644A>G (p.Asn215Ser)	VCV000010730.49	0.00003	Pathogenic
NM_000169.3(GLA):c.870G>A (p.Met.290Ile)	VCV000222435.20	0.000003	Pathogenic
NM_000169.3(GLA):c.937G>T (p.Asp313Tyr)	VCV000010738.87	Not available	VUS
NM_000169.3(GLA):c.352C>T (p.Arg118Cys)	VCV000042454.74	0.0006	Likely pathogenic

**Table A2:** Demographic and clinical characteristics of the Fabry disease patients.

Mutation, Phenotype Association n (%)	Group	Gender, Age	Birth City/Country	Family History	α-GAL Activity (pmol/h/spot)	Symptoms	Events	Risk Factors	RBBB	IVS (mm)	LGE Inferolateral
NM_000169.3(GLA):c.337T>C (p.Phe113Leu) Late-onset 6 (43%)	H	F, 60	VN de Famalicão	No	1.6	Dyspnea Chest pain	None	None	Yes	12	Yes
	H	M, 62	Porto	No	-	Fatigue	None	DLP	-	18	No
	H	M, 58	Angola	Yes	2.8	Fatigue Murmur	HF ICD	DLP CVD	Yes	22	Yes
	H	M, 66	Lisboa	No	-	Fatigue Dyspnea	HF	HTA	Yes	21	No
	H	M, 54	Guimarães	No	-	Fatigue Acroparesthesias; Angiokeratomes	None	DLP	No	16	Yes
	D	M, 74	Porto	No	-	Angiokeratomas	None	HTA	Yes	16	No
NM_000169.3(GLA):c.916C>T (p.Gln306Ter) Classic 1 (7.1%)	H	F, 67	VN de Famalicão	Yes	-	Dyspnea Murmur	None	DLP CVD	No	15	Yes
NM_000169.3(GLA):c.644A>G (p.Asn215Ser) Late on-set 1 (7.1%)	H	M, 43	Brazil	Yes	0.8	Palpitations Acroparesthesias	AF	Smoking	Yes	12	Yes
p. N215S + p.M290I Late on-set/classic 1 (7.1%)	D	F, 65	Vila Real	Yes	-	None	cAVB ICD	HTA DLP	Yes	13	Yes
NM_000169.3(GLA):c.870G>A (p.Met290Ile) Classic 3 (21%)	H	M, 56	VN de Gaia	Yes	3.0	Fatigue	Cardioembolic	HTA DLP	Yes	12	Yes
	D	M, 64	Vila Real	No	-	None	cAVB PM	HTA DLP CVD	No	10	-
NM_000169.3(GLA):c.937G>T (p.Asp313Tyr) Classic 1 (7.1%)	H	F, 63	Gondomar	Yes	-	Fatigue	Atrial Flutter	DM HTA CVD	No	12	No
NM_000169.3(GLA):c.352C>T (p.Arg118Cys) Late on-set 1 (7.1%)	D	F, 64	Coimbra	Yes	-	Fatigue Murmur	ICD	None	-	22	No
New (TBA) Classic 1 (7.1%)	H	F, 61	Penafiel	No	-	Fatigue Chest pain Edema	None	DM HTA DLP *Cornea verticillata*	No	16	Yes

AF, Atrial fibrillation; cAVB, complete Atrioventricular block; CVD, Cerebrovascular disease; D, dilated; DLP, dyslipidaemia; DM, diabetes mellitus; F, female; H, hypertrophic; HF, Heart failure; HTA, hypertension; ICD, Implantable cardioverter defibrillator; LGE, Late gadolinium enhancement; IVS, Interventricular septum; M, male; PM, pacemaker; RBBB, Right bundle branch block. “-” indicates data not available or not applicable.

**Table A3:** Comparison of demographics and clinical Fabry disease (FD) red flags between patients with the classical phenotype variants and patients with late-onset *GLA* mutations.

	Classical Phenotype n = 6	Late on-Set Variants n = 8
Age, median [range]	63.5 [56, 67]	62 [43, 74]
Male sex	2 (33%)	6 (75%)
LV Phenotype		
Dilated	2 (33%)	2 (25%)
Hypertrophic	4 (67%)	6 (75%)
Family history	4 (67%)	3 (38%)
At least one FD red flag	4 (67%)	5 (71%)
Arrhythmic events	3 (50%)	2 (25%)
Acroparesthesias	0 (0%)	2 (25%)
Angiokeratomas	0 (0%)	2 (25%)
*Cornea verticillata*	1 (17%)	0 (0%)
Cerebrovascular disease	3 (50%)	1 (13%)
At least one FD exam red flag	5 (83%)	7 (88%)
QRS, median [range]	164 [110, 169]	149.5 [105, 209]
QRS > 110	2 (67%)	3 (75%)
Right Bundle Branch Block	2 (33%)	4 (80%)
Fascicular block	2 (33%)	1 (20%)
Papillary hypertrophy	0 (0%)	1 (17%)
LGE Inferolateral	4 (80%)	4 (50%)

FD, Fabry disease; LGE, Late gadolinium enhancement; LV, Left ventricle.
