# Supplementary material for: Fabry Disease Screening in Patients with Idiopathic HCM or LVH: Data from the Multicentric Nationwide F-CHECK Study
Source: Biomedicines. 2025 Oct 16;13(10):2530. doi: 10.3390/biomedicines13102530 (PMC12561516; doi:10.3390/biomedicines13102530)
Supplement: Supplementary file 1 [file biomedicines-13-02530-s001.zip › biomedicines-3895702-supplementary.pdf]

STROBE Statement—checklist of items that should be included in reports of observational studies

	Item No.	Recommendation	Page No.	Relevant text from manuscript
**Title and abstract**	1	(*a*) Indicate the study's design with a commonly used term in the title or the abstract	1	"…multicenter observational study … of FD in a Portuguese cohort.."
		(*b*) Provide in the abstract an informative and balanced summary of what was done and what was found	1	
**Introduction**				
Background/rationale	2	Explain the scientific background and rationale for the investigation being reported	2	
Objectives	3	State specific objectives, including any prespecified hypotheses	3	"This study aims to screen for FD in patients presenting with a range of cardiac phenotypes … The objectives are to facilitate the timely diagnosis of FD, enhance understanding of the disease's national epidemiology, and raise awareness among clinicians managing patients whose clinical presentations may be attributable to FD."
**Methods**				
Study design	4	Present key elements of study design early in the paper	4	"The F-CHECK study was a multicentre, observational epidemiological study…"
Setting	5	Describe the setting, locations, and relevant dates, including periods of recruitment, exposure, follow-up, and data collection	4	"…conducted between January 2021 and January 2025, enrolling 409 patients referred from cardiomyopathy consultation across 10

				Portuguese hospitals… Patient recruitment commenced in April 2022, after obtaining ethical approval.”
Participants	6	(*a*) *Cohort study*—Give the eligibility criteria, and the sources and methods of selection of participants. Describe methods of follow-up<br>*Case-control study*—Give the eligibility criteria, and the sources and methods of case ascertainment and control selection. Give the rationale for the choice of cases and controls<br>*Cross-sectional study*—Give the eligibility criteria, and the sources and methods of selection of participants	4	“Inclusion criteria encompassed patients diagnosed with: idiopathic HCM, defined by LV wall thickness ≥ 15 mm (Group A); idiopathic LVH with wall thickness ≥ 13 mm (Group B); the dilated phase of HCM (Group C); and dilated cardiomyopathy of un-known aetiology, with late gadolinium enhancement (LGE) on cardiac magnetic resonance (CMR) affecting the inferolateral basal segment (Group D).”
		(*b*) *Cohort study*—For matched studies, give matching criteria and number of exposed and unexposed<br>*Case-control study*—For matched studies, give matching criteria and the number of controls per case	-	
Variables	7	Clearly define all outcomes, exposures, predictors, potential confounders, and effect modifiers. Give diagnostic criteria, if applicable	5	“Sociodemographic and clinical data were collected… including cardiovascular history, current and past symptoms and signs, cardiovascular risk factors, and medication use. Additionally, the most recent findings from biomarker assessments, electro-cardiogram (ECG), Holter

				monitoring, echocardiography, and CMR ...”
Data sources/ measurement	8*	For each variable of interest, give sources of data and details of methods of assessment (measurement). Describe comparability of assessment methods if there is more than one group	5	“Sociodemographic and clinical data were collected from electronic clinical records… Additionally, the most recent findings from electrocardiogram (ECG), Holter monitoring, echocardiography, and CMR…”
Bias	9	Describe any efforts to address potential sources of bias	4,5	“Variant classification and description were performed by the certified laboratories … reducing subjective classification bias… The study included all consecutive patients meeting inclusion criteria during the recruitment period across participating centers to minimize sources of bias …The relatively small number of FD cases, particularly in subgroup analyses, is acknowledged as a limitation and discussed …”
Study size	10	Explain how the study size was arrived at	5	“No formal sample size calculation or power analysis was performed a priori… Consequently, the sample size was determined by patient availability rather than statistical considerations.”

Continued on next page

Quantitative variables	11	Explain how quantitative variables were handled in the analyses. If applicable, describe which groupings were chosen and why	5	"Variables are presented as median and interquartile range (IQR)… Mann-Whitney U test was applied to continuous variables."
Statistical methods	12	(*a*) Describe all statistical methods, including those used to control for confounding	5	"The Mann-Whitney U test was applied to continuous variables, while categorical variables were analysed using the appropriate chi-square test or Fisher's exact test… Associations between FD and clinical or imaging features were assessed using logistic regression..."
		(*b*) Describe any methods used to examine subgroups and interactions	5	
		(*c*) Explain how missing data were addressed	5	"Missing values were reported explicitly in tables using "-" where data were not available. No imputation was performed. Analyses were conducted using available-case data for each variable."
		(*d*) *Cohort study*—If applicable, explain how loss to follow-up was addressed<br>*Case-control study*—If applicable, explain how matching of cases and controls was addressed<br>*Cross-sectional study*—If applicable, describe analytical methods taking account of sampling strategy		
		(*e*) Describe any sensitivity analyses		
**Results**				
Participants	13*	(a) Report numbers of individuals at each stage of study—eg numbers potentially eligible, examined for eligibility, confirmed eligible, included in the study, completing follow-up, and analysed	5	"A total of 409 patients were enrolled in the study…"
		(b) Give reasons for non-participation at each stage		
		(c) Consider use of a flow diagram	4	

Descriptive data	14*	(a) Give characteristics of study participants (eg demographic, clinical, social) and information on exposures and potential confounders	5-9	"A total of 409 patients were enrolled in the study, including 250 males (61%), with a median age of 64 (range:18-93) years…"
		(b) Indicate number of participants with missing data for each variable of interest	5-9	
		(c) *Cohort study*—Summarise follow-up time (eg, average and total amount)		
Outcome data	15*	*Cohort study*—Report numbers of outcome events or summary measures over time		
		Case-control study—Report numbers in each exposure category, or summary measures of exposure		
		Cross-sectional study—Report numbers of outcome events or summary measures	5-9	
Main results	16	(*a*) Give unadjusted estimates and, if applicable, confounder-adjusted estimates and their precision (eg, 95% confidence interval). Make clear which confounders were adjusted for and why they were included	9-10	"Logistic regression analyses were performed to evaluate associations between FD and clinical as well as imaging features (Table 5). For the overall cohort, both unadjusted and multivariable-adjusted ORs with 95% CIs were calculated, adjusting for age, sex, comorbidities, and medication use…"
		(*b*) Report category boundaries when continuous variables were categorized	6,8,9	"QRS > 110ms"
		(*c*) If relevant, consider translating estimates of relative risk into absolute risk for a meaningful time period		

Continued on next page

Other analyses	17	Report other analyses done—eg analyses of subgroups and interactions, and sensitivity analyses		
**Discussion**				
Key results	18	Summarise key results with reference to study objectives	11-13	"The identification of seven distinct GLA gene mutations in a cohort of 409 cardiomyopathy patients, yielding an approximate prevalence of 3.4%, suggests that a substantial number of FD cases within cardiac populations may remain undiagnosed through conventional clinical pathways or broader screening efforts…"
Limitations	19	Discuss limitations of the study, taking into account sources of potential bias or imprecision. Discuss both direction and magnitude of any potential bias	11-13	"…Nevertheless, the wide CIs observed across several ORs reflect the small number of FD patients, introducing statistical uncertainty despite consistent effect directions… underscoring the challenges of regression analysis in rare diseases. These findings highlight the need for larger, multicentric studies to validate the identified associations and refine the phenotypic predictors of FD across the hypertrophic–dilated spectrum."
Interpretation	20	Give a cautious overall interpretation of results considering objectives, limitations, multiplicity of analyses, results from similar studies, and other relevant evidence	13	
Generalisability	21	Discuss the generalisability (external validity) of the study results	13	
**Other information**				
Funding	22	Give the source of funding and the role of the funders for the present study and, if applicable, for the	13	

	original study on which the present article is based

*Give information separately for cases and controls in case-control studies and, if applicable, for exposed and unexposed groups in cohort and cross-sectional studies.

Note: An Explanation and Elaboration article discusses each checklist item and gives methodological background and published examples of transparent reporting. The STROBE checklist is best used in conjunction with this article (freely available on the Web sites of PLoS Medicine at http://www.plosmedicine.org/, Annals of Internal Medicine at http://www.annals.org/, and Epidemiology at http://www.epidem.com/). Information on the STROBE Initiative is available at www.strobe-statement.org.
